# Pedicle screw-rod fixation: a feasible treatment for dogs with severe degenerative lumbosacral stenosis

**DOI:** 10.1186/s12917-015-0614-3

**Published:** 2015-12-07

**Authors:** Anna R. Tellegen, Nicole Willems, Marianna A. Tryfonidou, Björn P. Meij

**Affiliations:** Department of Clinical Sciences of Companion Animals, Faculty of Veterinary Medicine, Utrecht University, Yalelaan 108, 3584 CM Utrecht, The Netherlands

**Keywords:** Lumbosacral fixation, Pedicle screws, Degenerative lumbosacral stenosis, Intervertebral disc degeneration, Dog, Discospondylitis

## Abstract

**Background:**

Degenerative lumbosacral stenosis is a common problem in large breed dogs. For severe degenerative lumbosacral stenosis, conservative treatment is often not effective and surgical intervention remains as the last treatment option. The objective of this retrospective study was to assess the middle to long term outcome of treatment of severe degenerative lumbosacral stenosis with pedicle screw-rod fixation with or without evidence of radiological discospondylitis.

**Results:**

Twelve client-owned dogs with severe degenerative lumbosacral stenosis underwent pedicle screw-rod fixation of the lumbosacral junction. During long term follow-up, dogs were monitored by clinical evaluation, diagnostic imaging, force plate analysis, and by using questionnaires to owners.

Clinical evaluation, force plate data, and responses to questionnaires completed by the owners showed resolution (*n* = 8) or improvement (*n* = 4) of clinical signs after pedicle screw-rod fixation in 12 dogs. There were no implant failures, however, no interbody vertebral bone fusion of the lumbosacral junction was observed in the follow-up period. Four dogs developed mild recurrent low back pain that could easily be controlled by pain medication and an altered exercise regime.

**Conclusions:**

Pedicle screw-rod fixation offers a surgical treatment option for large breed dogs with severe degenerative lumbosacral stenosis with or without evidence of radiological discospondylitis in which no other treatment is available. Pedicle screw-rod fixation alone does not result in interbody vertebral bone fusion between L7 and S1.

## Background

Low back pain in dogs is a common clinical problem and can be the result of several pathologies [1]. Degenerative lumbosacral stenosis (DLSS) is the most common cause of caudal lumbar back pain in middle to large breed dogs [2]. DLSS is characterized by bony and soft tissue changes leading to stenosis of the spinal canal and moderate to severe compression of the cauda equina. The intervertebral disc (IVD) is often degenerated and this results in a shift of load bearing from the IVD to surrounding structures. This may lead to spinal instability [2]. Low back pain can also be caused by other conditions, such as discospondylitis [3], trauma (fracture and/or luxation), or neoplasia [3, 4]. Discospondylitis is a bacterial infection of the IVD and adjacent intervertebral end plates and commonly originates from a primary urogenital infection via haematogenous spread [3]. Discospondylitis can result in severe proliferation of fibrous tissue and bone, vertebral instability, subchondral bone resorption and secondary DLSS [5]. Computed tomography (CT) and magnetic resonance imaging (MRI) are the most informative modalities to investigate the LS area [6, 7].

Treatment of DLSS can be conservative or surgical. Low back pain in DLSS can be treated with non-steroidal anti-inflammatory drugs and/or opioid analgesics, body weight reduction, and an adjusted exercise pattern or physiotherapy. Epidural infiltration with methylprednisolone acetate has been reported as medical treatment for DLSS provided that the dog does not show urinary or faecal incontinence and proprioceptive deficits, and does not suffer from concurrent discospondylitis [8]. In case of discospondylitis long term antibiotic drugs are the primary treatment. Surgical treatment of DLSS is accomplished by dorsal laminectomy or foraminotomy, and if indicated, partial discectomy and uni- or bilateral facetectomy. In the short-term, surgical intervention leads to improvement of clinical signs in 78–93 % of cases [9, 10] but in the long-term clinical signs recurred in 17–38 % of cases [9, 10], which is also known as failed back syndrome [11, 12]. Moreover, force plate analyses (FPAs) showed that the propulsive force of the pelvic limbs is not fully restored after decompressive surgery for DLSS [13]. It has been postulated that decompressive surgery, and especially facetectomy, can worsen LS instability in some patients, resulting in further overall degeneration and recurrence of worsening of clinical symptoms [9, 14].

Therefore, we previously investigated the feasibility of pedicle screw-rod fixation (PSRF) in a cadaver study [14, 15] and in an in vivo pilot study [14] in large breed dogs. Screw entry points and guideline values for safe insertion of pedicle screws into the canine L7 and S1 vertebrae have been determined in other studies [14, 16, 17]. The purpose of spinal fixation and interbody fusion is to restore and maintain disc space height and to increase the stability of the operated segment [18], thereby making further ongoing degenerative changes clinically irrelevant. The aim of the present study is to report the long term results of PSRF in 12 client-owned dogs with severe DLSS and also to assess whether PSRF leads to spinal fusion of the LS junction.

## Results

### Dogs

Seven male (3 intact, 4 neutered) and five female (2 intact, 3 neutered) dogs with a median age of 8 years (1–12 years) and a median body weight of 32 kg (22–55 kg) were included in the study (Table 1). All dogs were kept as companion animals. Four dogs had undergone decompressive surgery previously but developed failed back syndrome.

**Table 1:** Overview of signalment, history and radiological diagnosis in 12 dogs with lumbosacral degenerative stenosis (DLSS) and/or discospondylitis that were treated with pedicle screw-rod fixation

Dog	Breed	Sex	Age (yrs)	History	Radiological diagnosis
1	Labrador retriever	FC	5	LS pain, paraparesis	DLSS & DS
2	Rottweiler	M	8	LS pain	DLSS & DS
3	GSD	FC	8	LS pain, paraparesis	DLSS & DS
4	GSD	MC	11	LS pain, paraparesis; DL 6 yrs earlier	DLSS & DS
5	Rhodesian Ridgeback	F	10	LS pain, left paraparesis, urinary incontinence; DL 6 months earlier	DLSS & DS
6	GSD	M	12	LS pain, paraparesis	DLSS & DS
7	Cane Corso	MC	7	LS pain	DLSS
8	American Bulldog	M	5	LS pain	DLSS & DS
9	Border Collie	MC	9	LS pain	DLSS & DS
10	Rhodesian Ridgeback	FC	7	LS pain, paraparesis; DL 4 yrs earlier	DLSS
11	Vizsla	MC	12	LS pain	DLSS
12	American Staffordshire Bull Terrier	F	5	LS pain, left paraparesis; DL 3 yrs earlier	DLSS

*Abbreviations: LS* lumbosacral, *GSD* German Shepherd dog, *F* female, *FC* female castrated, *M* male, *MC* male castrated, *DS* discospondylitis, *DL* dorsal laminectomy, *yrs* years

### Clinical examination

All dogs presented with pelvic limb lameness and caudal lumbar pain; seven dogs also showed paraparesis. In all dogs pain was evoked upon pressure and extension of the LS spine and tail extension. One dog suffered from urinary incontinence. The neurological Griffith score before surgery was grade 1 (5 dogs), 2 (4 dogs) and 3 (3 dogs) (Table 2).

**Table 2:** Overview of surgery details and clinical outcome in 12 dogs with lumbosacral degenerative stenosis (DLSS) that were treated with pedicle screw-rod fixation

Dog	Surgery	Bone graft	Clinical outcome (follow-up period)	Griffith score	
				pre-op	last FU
1	L7-S1: DL, PD, PSRF	Bone L7 + S1	Excellent (4 yrs)	3	0
2	L7-S1: DL, PD, PSRF	Bone L7 + S1	Excellent (4 yrs)	1	0
3	L7-S1: DL, PD, PSRF	Bone L7 + S1	Excellent (3 yrs)	2	0
4	L7-S1: rDL, PD, PSRF & Distraction	Bone iliac crest. Osteostixis EPs	Improved (euth. 6 mo, heart disease)	2	2
5	L7-S1: rDL, PD, L Facetectomy, L Foraminotomy, Excision L7 nerve, PSRF	Bone L7 + S1	Improved (euth. 15 mo, neoplasia)	3	3
6	L6-S1: DL, L7-S1: DL, PSRF	Bone L7 + S1	Improved (1.5 years, euth. hemangiosarcoma)	2	0
7	L7-S1: DL, PD, L&R Facetectomy, PSRF & Distraction	Bone L7 + S1	Excellent (1 yr)	1	0
8	L7-S1: DL, PD, PSRF	Bone L7 + S1 Burring EPs	Improved (euth. 8 mo)	3	1
9	L6-S1: DL, L7-S1: PD; L&R Facetectomy, PSRF & Distraction	Bone L7 + S1	Excellent (6 mo)	1	0
10	L7-S1: rDL, PD, PSRF	Bone L7 + S1	Excellent (6 mo)	2	0
11	L6-S1: DL, L7-S1: PD	Bone L7 + S1	Excellent (11 mo)	1	0
	PSRF & Distraction				
12	rDL, Partial L Facetectomy, L Foraminotomy, PSRF	None	Excellent (6 mo)	1	0

Excellent: resolution of clinical signs. Improved: decrease of clinical signs

*Abbreviations*: *DL* dorsal laminectomy, *rDL* revision DL, *PD* partial discectomy, *PSRF* pedicle-screw rod fixation, *L* left, *R* right, *EPs* end plates, *yrs* years, *mo* months, *euth.* euthanized, *FU* follow-up

### Diagnostic imaging

**Fig. 1:**
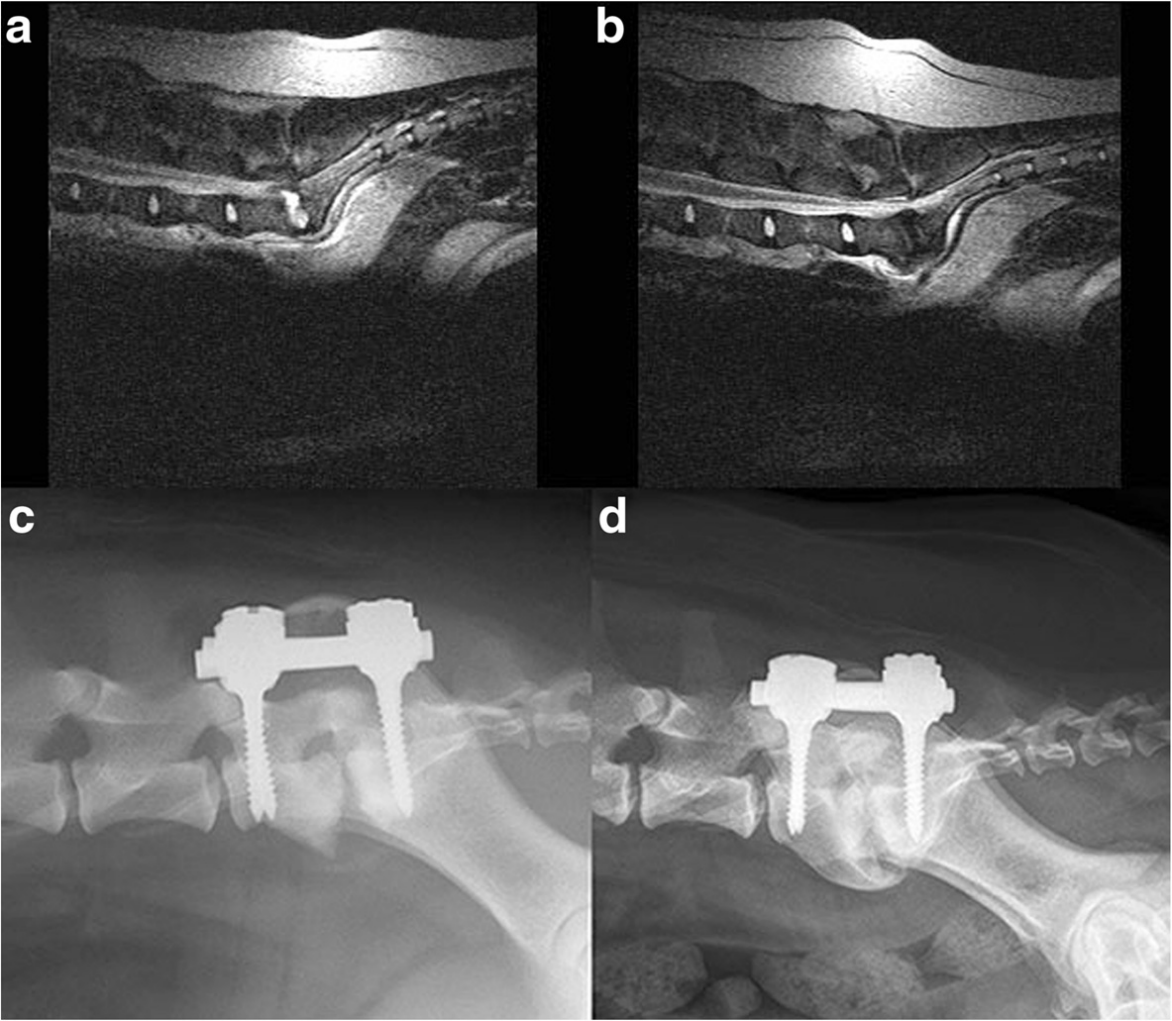
**a** Sagittal T2-weighted MR image of a 5-year-old Labrador retriever (dog 1) with degenerative lumbosacral stenosis and acute onset of discospondylitis. There is a hyperintense signal (exudate) visible in the intervertebral disc space. **b** Sagittal T2-weighted MR image of dog 1 after three months of treatment with oral antibiotics. The inflammatory exudate has disappeared. **c** Immediate postoperative radiograph of dog 1 after pedicle screw-rod fixation (PSRF) showing osteolysis of the L7 and S1 endplates. **d** Radiograph of dog 1 at four years after PSRF. Spondylosis deformans has formed ventral to the LS junction

**Fig. 2:**
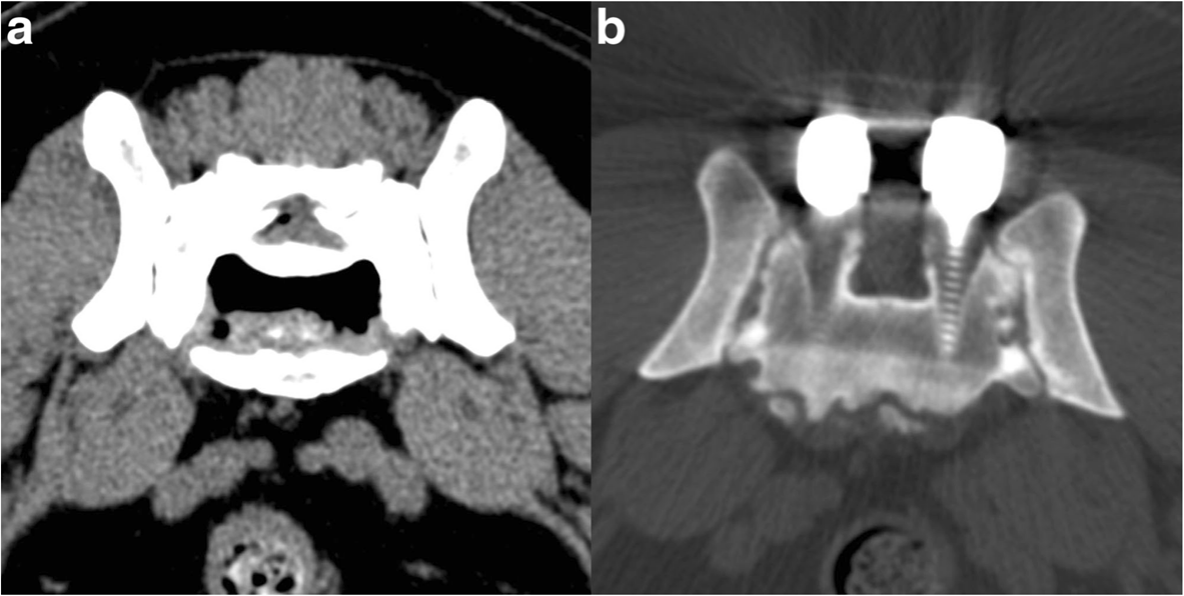
**a** Transverse CT image of the lumbosacral (LS) junction of a 9-year-old Border collie (dog 9) with degenerative lumbosacral stenosis and discospondylitis. Spinal stenosis and severe intervertebral disc (IVD) bulging are visible and there is gas accumulation (vacuum phenomenon) present in the center of the L7-S1 IVD. **b** Transverse CT image at the level of S1 of a dog (dog 1) with pedicle screw-rod fixation, four years after implantation. No bony fusion between the L7 and S1 vertebrae was visible

**Fig. 3:**
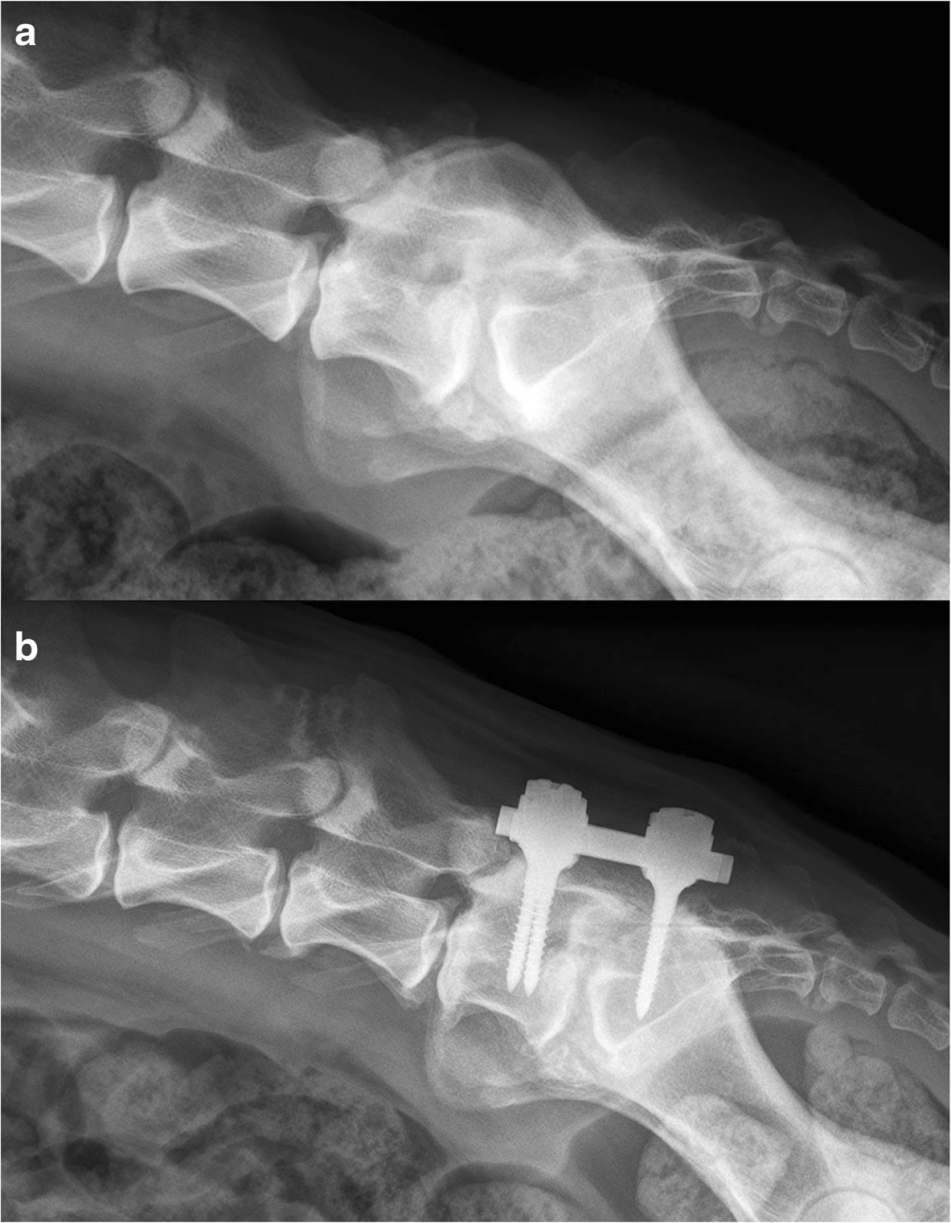
**a** Pre-operative radiograph of an 8-year-old German shepherd dog (dog 3) diagnosed with degenerative lumbosacral stenosis. There is non-bridging spondylosis deformans, end plate sclerosis, lumbosacral step formation and elongation of the sacral lamina underneath L7. **b** Radiograph showing dog 3 three years after pedicle screw-rod fixation with implants in correct position. At the level of L5-L6 and L6-L7, there is radiological evidence for adjacent segment pathology, seen by narrowing of the intervertebral foramen. No interbody fusion was present between L7 and S1

Imaging was performed pre-operatively using plain radiography (4 dogs), CT (12 dogs), and MRI (5 dogs) (Table 3). In all 12 dogs the final radiological diagnosis was DLSS with presumptive radiologic evidence of concurrent discospondylitis in eight dogs (Table 3). Pre-operative radiologic- and CT findings included spinal stenosis of the lumbosacral junction (Fig. 2a) in ten dogs, end plate sclerosis of both lumbosacral end plates (Fig. 3) in eleven cases, end plate osteolysis (Fig. 1) in seven cases, vacuum phenomenon in the IVD (Fig. 2a) in three cases, elongation of the sacral lamina up to or under the caudal end of the lamina of L7 as described by Suwankong et al. [6] (Fig. 1) in four cases and LS step formation (ventral subluxation of S1 with respect to L7) (Fig. 3) in four cases. A narrowed IVD space was visible in two dogs. Non-bridging spondylosis deformans (Fig. 3) was recorded pre-operatively in nine dogs, bridging spondylosis in two dogs. Protrusion of the IVD was seen in all dogs; severe protrusion (>50 % reduction of spinal canal width) (Fig. 1a) in ten dogs, a moderate compression (25–50 % reduction of spinal canal width) in one dog and mild protrusion (<25 % reduction of spinal canal width) in one dog. Dorsal displacement of the dural sac, combined with a decrease in the epidural fat signal dorsal to the dural sac at the level of L7-S1 was recorded in nine dogs on MRI or CT (Fig. 1). Thickening of spinal nerves was detected in four dogs. The signal intensity of the L7-S1 IVD on T2-weighted images was severely decreased in all five dogs which underwent MRI (Fig. 1). Dog 4 had undergone dorsal laminectomy 6 years earlier (Table 1) and on MR a bulging LS disc was noted in combination with dorsal displacement of nerve tissue at the level of L7-S1. Calcifications in the IVD space were recorded as well. On the CT images there was marked ventral spondylosis deformans, IVD calcifications and vacuum phenomenon. Moreover, there was still severe central and right lateral disc protrusion present, leading to the right lateral nerve compression near the right facet joint. Dog 5 had undergone decompressive surgery 6 months earlier (Table 1). CT showed that the cauda equina was displaced dorsally as a consequence of bulging disc material. Both the L7 and S1 end plates were irregular and sclerotic. There was pronounced new bone formation around the lumbosacral junction, in the intervertebral foramina and around the sacroiliac joints. The left exiting spinal nerve was markedly enlarged, indicative for a peripheral nerve sheath tumor. There was severe muscle atrophy present in the left quadriceps and gluteus muscles.

**Table 3:** Overview of read out parameters in 12 dogs with lumbosacral degenerative stenosis (DLSS) treated with pedicle screw-rod fixation

Dog	Pre-op	Intra-op	Post-op	Follow up period (months)		
				< 3 mo	< 6 mo	> 6 mo
1	CT, MRI	BC, HP	RX	CT		CT, RX (12 mo); CT, RX, FPA (46 mo)
2	RX, CT	BC, HP	RX	RX		CT, FPA (40 mo)
3	RX, CT	BC, HP	RX	RX		RX, FPA (35 mo)
4	CT, MRI	BC, HP	RX, CT	RX		
5	CT, MRI	BC, HP	RX			
6	RX, CT	BC	RX		RX	
7	CT, MRI		RX			CT, FPA (10 mo)
8	CT	BC	RX	CT, RX, FPA	CT, RX, FPA	CT, FPA (7,5 mo)
9	CT	BC, HP	RX	RX		
10	CT, MRI	BC, HP	RX	RX, FPA	CT, FPA	
11	CT, RX, MRI		RX	RX	RX, CT	
12	CT	BC, HP	RX	RX	RX, FPA	CR (14 mo)

*Abbreviations*: *Pre-op* pre-operative, *intra-op* intra-operative, *post-op* postoperative, *mo* months, *CT* computed tomography, *MRI* magnetic resonance imaging, *BC* bacteriologic culture, *HP* histopathologic evaluation, *RX* plain radiography, *FPA* force plate analysis

### Surgical findings

Following dorsal laminectomy and partial discectomy (Table 2), pedicle screws were inserted and were used to distract, realign and stabilize the LS segment. In ten dogs, the protrusion of the IVD was considered severe, in two dogs, there was moderate protrusion. The amount of epidural fat was decreased in ten dogs and absent in one dog. In six dogs, inflammation of the epidural fat was noticed by the surgeon. In 11 dogs thickening of neural tissue, especially the S1 nerve roots, was visible.

Two of ten disc tissue samples returned with a positive bacterial culture. *Bacillus spp* (dog 5) and *Staphylococcus aureus* (dog 8) were identified in two dogs.

Histopathological examination of tissue samples collected during surgery showed degeneration of the annulus fibrosus and nucleus pulposus in all cases. Histopathological examination of the excised nerve (dog 5) showed an undifferentiated neurofibrosarcoma of the nerve root, characterized by round- and spindle shaped neoplastic cells.

### Follow-up (imaging and clinical signs)

Radiography or CT was performed to evaluate the position of the screws and the amount of interbody vertebral bone fusion. In the follow-up period after surgery imaging was performed at 4–6 weeks (radiography or CT, 7 dogs), at three months (radiography or CT, 4 dogs), at six months (CT, 3 dogs), at one year (CT, 2 dogs), at three years (radiography or CT, 2 dogs), and at four years (CT, 1 dog) (Table 2).

Placement of the screws was considered to be correct [14] in 11 out of 12 dogs (92 %) based on radiographic evaluation. In six dogs, CT was performed postoperatively (Fig. 1b). Optimal screw anchorage was achieved by involving both the medial and lateral pedicle cortex. Cortical encroachment of the lateral pedicle wall was noticed on CT with the right L7 screw in two dogs. Penetration of the ventral cortex was recorded on CT in three dogs, involving four screws. No implant failures were seen.

In eight dogs, there was complete resolution of clinical signs after surgery, in two dogs the severity of the clinical signs decreased. These two dogs (dog 4 and 5) had already undergone prior decompressive surgery by dorsal laminectomy. In two dogs (dog 6 and 8), the clinical signs recurred after initial remission. Plain radiographs and CT scans were obtained. No adverse advents as a result of the pedicle screw implantation surgery were noted. Neurologic dysfunction in dog 6 did not improve markedly after surgery and dragging with the left hind limb persisted. Dog 8 was euthanized at eight months after surgery at request of the owner, since low back pain recurred every time antibiotic treatment was ceased.

After surgery, the Griffith neurological grading score was 0 (9 dogs), 1 (1 dog), 2 (1 dog) and 3 (1 dog) (Table 1). The median pre-operative Griffith score was 2 (with a range from 1–3), whereas the Griffith score obtained at the last follow up visit was 0 (with a range from 0 to 3). The Wilcoxon signed rank test revealed a significant improvement in Griffith scores before surgery and at the last follow up visit (*p* = 0.004).

The development of adjacent segment pathology (ASP) was noticed in one dog after three years on plain radiographs (Fig. 3), but the dog did not display signs of low back pain. At any time point after PSRF, in none of the other dogs ASP was noticed on diagnostic imaging nor clinically.

In the four dogs that underwent manual distraction of the LS junction, the IVD space height increased by 67 % (dog 4), 11 % (dog 7), 114 % (dog 9), and 9 % (dog 11) compared with the IVD height prior to surgery. Six months after surgery, distraction of the LS junction was still present in three dogs. In one dog (dog 11) there was loss of distraction as evidenced by sudden low back pain at one week postoperatively, and radiographic evidence of collapse of the L7-S1 IVD space without implant failure. The pain was controlled with oral analgesics for two weeks.

### Force plate analysis

**Fig. 4:**
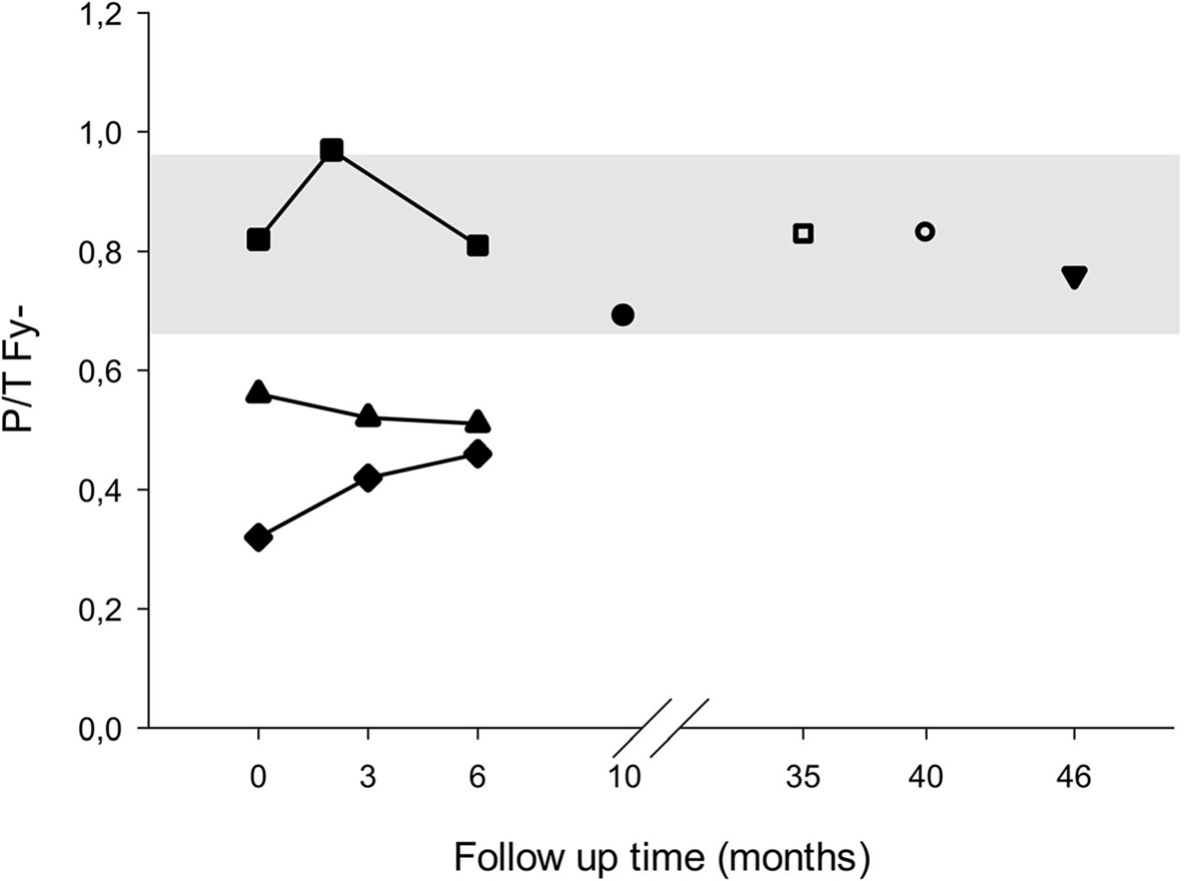
P/T Fy- values of seven dogs (■ = dog 12; ▲ = dog 10; ♦ = dog 8; ● = dog 7; □ = dog 3; ○ = dog 2; ▼ = dog 1). The grey area marks reference values for the average P/T Fy- value ±1 SD previously determined for healthy dogs [19]

Pre-operative force plate analysis (FPA) was performed in three dogs (dogs 8, 10 and 12) (Fig. 4). In two dogs (dogs 8 and 10), the P/T Fy- and P/T Fz + ratios were lower than reference ranges described in a previous study [19]. In dog 10, FPA was performed six months after surgery and values were still below reference ranges, this dog was lost for further follow up. Dog 7 showed normal P/T Fy- values after 10 months. FPA performed in three dogs (dog 1, 2 and 3) more than three years after surgery showed P/T Fy- ratios comparable to normal dogs [19].

### Owner questionnaires

Eight out of twelve (67 %) owners responded to the questionnaire. The follow-up period ranged from five months to more than four years. Prior to surgery, owners mentioned low back pain, hind limb lameness, and reluctance to perform certain movements as the most striking clinical signs. All owners reported that the clinical signs of their dog had disappeared after surgery. However, in four dogs the clinical signs recurred. In three dogs these signs were mild and could be treated effectively with NSAIDs. In the fourth dog, discospondylitis persisted despite aggressive long term (6 months) antibiotic therapy, for which the dog was eventually euthanized at eight months after PSRF. Six owners did not report any recurrences during the follow-up period; the long term outcome of three dogs is unknown since they were euthanized due to unrelated illnesses (i.e. heart failure, hemangiosarcoma). Three dogs showed concurrent orthopedic problems such as hip dysplasia (*n* = 1) and osteoarthritis of the stifle joint (*n* = 2) in the follow up period. Five dogs continued to receive intermittent pain medication, with four dogs receiving non-steroidal anti-inflammatory drugs (NSAIDs), one dog tramadol and one dog a combination of NSAIDs and neuromodulatory drugs (gabapentin).

Before evaluating the answers of the owners to the questionnaire, the reliability of the answers was tested by calculating the Cronbach’s alpha value. The Cronbach’s alpha value of the responses to the questionnaire was 0.88, indicating that the answers were reliable. The data were normally distributed. All eight owners that had filled in the questionnaire reported that the clinical signs of low back pain had disappeared after surgery (100 %). Three owners reported that the clinical signs of low back pain had recurred after an asymptomatic period of time (3/8 = 38 %). Table 4 shows the results to the questionnaire before, six months, and more than one year after surgery. All data are expressed as the median and the range. The level of significance was set at *P* <0.05. There was a significant and sustained decrease in caudal limb lameness, caudal lumbar pain and difficulty in rising up more than six months after surgery. Moreover, muscle volume had significantly increased six months after surgery, compared to the pre-operative situation. There was a trend of decrease in pelvic hind limb lameness and hypersensitivity of the caudal spine (*P* = 0.061) after six months.

**Table 4:** Results (median and range) of responses to the questionnaires of dogs treated with PSRF before surgery, after 6 months and more than 1 year after surgery

Questions	Before surgery	After 6 months	After more than 1 year
Complaints of pelvic limbs	3 (1–4)	8 (4–10)^a^	7 (5–9)^a^
Pelvic limb weakness	4 (1–10)	8 (6–10)^b^	7 (6–10)^b^
Caudal lumbar pain	1 (1–4)	7 (4–10)^a^	7 (5–9)^a^
Difficulty rising up	4 (1–6)	8 (7–10)^a^	7 (5–10)^a^
Difficulty lying down	8 (1–6)	10 (8–10)	10 (5–10)
Muscle volume of the pelvic limbs	4 (1–7)	7 (5–9)^a^	7 (6–7)^a^
Position of the tail	3 (1–10)	9 (1–10)	9 (1–10)
Movement of the tail	5 (2–10)	10 (3–10)	9 (3–10)
Control of urination and defecation	10 (3–10)	10 (10–10)	10 (10–10)
Hypersensitivity of the caudal spine	3 (1–10)	9 (3–10)^b^	10 (9–10)

^a^ *P* < 0.05, Friedman test, compared with value before surgery. ^b^Group comparisons were borderline significant (*P* = .061), individual comparisons of time points did show significant difference (*P* < 0.05)

## Discussion

The pilot study of Smolders et al. [14] suggested that PSRF of the canine LS junction can be used as an addition to surgical decompression for dogs with LS disease and presumed instability of the LS joint. The results showed stability of the implants and improvement of hind limb function in Greyhounds with mild LS disease. The current study presented the follow-up of 12 client-owned dogs with severe DLSS treated with PSRF. With data retrieved from diagnostic imaging, FPAs and clinical examinations together with owner questionnaires, we conclude that PSRF can be a feasible treatment option for dogs with DLSS in which previous decompressive surgery failed and/or medical treatment is ineffective to control low back pain. The authors are aware of the limitations of this retrospective study. The study group is relatively small and due to the retrospective nature of the study, the follow up of the patients was not standardized. Not all owners were willing to attend control visits with their dog or had financial constraints. All cases were referred as severe and complicated cases, where conservative treatment or previous surgery had failed, or for which no other treatment was available. Even more, in several cases euthanasia was advised by the referring veterinarian but the owner persisted for third opinion referral.

Propulsive forces in the hind limb are decreased in dogs with DLSS as compared to healthy dogs [19]. In the present study, the collected FPA data showed an initial worsening after surgery, but after six months overall results were improving, with values at six months after surgery higher than before surgery. Notably, ground reaction forces were comparable to normal dogs (Table 3) [19]. These findings are in agreement with results from previous studies on FPA before and after decompressive surgery [19] and the in vivo pilot study on PSRF from the same group [14]. Given that FPA is used to objectively measure ground reaction forces in both humans and dogs [19–22], these findings indicate an overall clinical improvement in the long term.

The percentages of dogs with clinical remission and recurrence found in our study were similar to those for dorsal laminectomy alone [19], although the dogs in the current study suffered from more severe LS disease than the average population undergoing decompressive surgery. Decompressive surgery has proven to be insufficient in a small percentage of cases, i.e., due to the development or worsening of LS instability after surgery [9, 10, 14]. In four dogs in this study a previous decompressive surgery was already performed with inadequate effect. In humans with low back pain due to end stage degenerative disc disease, spinal fusion using cages with or without pedicle screw fixation is currently the state-of-the-art [23–25], rather than decompressive surgery alone. Moreover, spinal fusion is often performed during revision surgery for failed back syndrome [12, 26, 27].

Placement of the screws was considered to be correct [14] in 48/52 screws (92 %) and no implant failures were seen. In three cases, cortical encroachment of the medial pedicle wall by four screws was detected but this did not result in clinical signs. Optimal screw anchorage is achieved by involving the cis- and trans-cortex, as well as the medial and lateral pedicle wall. Full penetration of the ventral cortex was seen with seven screws in five dogs. Although full penetration carries the risk of damaging vascular structures, there was no indication that this happened. Full penetration is most likely the result of the fixed length of the screws. The PSRF device that was used in this study was produced for paediatric human spinal fixation which apparently was still too large for some of the dogs, e.g. dog number 11 (Border collie). This underscores the need for the development and production of pedicle screws for the canine species.

The aim of PSRF is to stabilize the LS junction. This is achieved in the short term by the inserted instrumentation and in the long term by fusion of the spinal segments as well. However, in this study no interbody vertebral bone fusion was achieved. Several authors have reported on surgical stabilization in the veterinary field as well. Mckee et al. [28] have performed distraction-stabilization in dogs with discospondylitis by the method described by Slocum et al. [29] and Auger et al. [30] have performed articular facet joint distraction with an external fixator. More recently, Golini et al. published a study about transarticular fixation as treatment for DLSS in dogs [31]. In all abovementioned studies, a considerable number of implant failures was seen which in some cases required additional surgery. In the current study, there was no implant failure. The dogs recovered very well but there was no evidence for spinal fusion in the long term follow-up. To achieve interbody fusion, additional methods are necessary. In the current study, we used autologous bone grafts in 11 cases but without success as far as bony fusion is concerned. Fitzpatrick and colleagues developed a dorsal fixation system, which uses a screw-rod construct in combination with a wedge-shaped screw. This screw is positioned in between the L7 and S1 vertebrae [32, 33]. With this device, bone ingrowth was visible. In human medicine, interbody spinal fusion is promoted by several techniques. In addition to iliac crest autograft, metal and composite interbody cages, allograft bone dowels and bone grafts infused with recombinant bone morphogenetic proteins (BMPs) or bone marrow derived stem cells are readily available for human patients and show promising effects [18, 34]. Moreover, in dogs the subchondral bone is relatively thicker than in humans whereas the canine end plates are thinner [35, 36]. This may counteract bony fusion between the two vertebrae in canines.Therefore more aggressive burring of the end plates to penetrate the subchondral bone would be appropriate in canines to achieve spinal fusion. Although bony fusion of the last lumbar vertebra and the sacrum is desired, there was no significant difference in outcome in human [27] and canine [30] patients that did show spinal fusion compared to patients that failed to develop interbody fusion after spinal fusion surgery [27, 30].

Recurrence of clinical signs after PSRF stabilization could be related to ASP. ASP can be defined as degeneration or other pathologic processes occurring cranial or caudal to a region of vertebral column fusion, the most common pathology being IVD degeneration [37]. In the current study, only two vertebrae were fixated. One of the dogs (dog 3) in this study showed signs of ASP on radiography at three years after surgery. This dog was not painful on the lumbar region during clinical examination and also the force plate data showed no signs of lumbar pain. ASP has been found in humans after spinal fusion surgery [38] and also in dogs after cervical fusion [39, 40]. Lumbar spinal fusion in humans resulted in radiologic evidence of ASP in 10–80 % after 10 years. Loss of motion in the fused segment leads to increased workload and altered biomechanics in adjacent segments [41]. However, at this moment it is unclear if ASP is a natural degenerative process or if ASP is the result of fusion surgery [42]. Clinically relevant ASP was only noted in 6–26.1 % of the human patients, with radiologic confirmed ASP, after ten years [41]. ASP does not seem a frequent clinical problem in dogs, most likely since they may not live long enough to develop ASP. In humans, the increasing number of fused vertebrae is associated with an increased risk of developing ASP. Additionally, a dorsal laminectomy adjacent performed to the fused segment, pre-existent IVD degeneration and pre-existent facet degeneration in the adjacent segment are also risk factors associated with the development of clinical ASP [41, 43].

Only two of the ten bacterial cultures showed a positive result, even though in eight dogs, there was radiological evidence for discospondylitis. It remains also unclear whether in these eight dogs discospondylitis was the primary etiology or whether it was superimposed on pre-existent DLSS since the end stage of severe lumbosacral discospondylitis is usually DLSS. Making the definitive diagnosis of discospondylitis is also challenging, for the detection of bacteria in the IVD can be rather difficult. Extensive degenerative changes in the IVD could also resemble discospondylitis. Urine and blood cultures only give positive results in 29 to 78 % of the cases [44, 45] and due to antibiotic treatment prior to culture, bacterial cultures often remain negative [45, 46]. This could also be the case in our study, as five dogs were treated for discospondylitis conservatively with antibiotics prior to the collection of disc material for bacterial culture. Interestingly, the topic of bacteria in IVDs causing low back pain has received considerable attention in recent years in the field of spine research in humans and has since been the subject of heated debate [47–49]. This debate was initiated by reports by Albert et al. [50] on findings of bacteria in IVD material harvested during spinal surgery [51] and publication of a randomized clinical trial showing successful treatment of humans with chronic low back pain using long term oral antibiotics [52]. In the light of these findings in humans, the positive bacterial cultures in our canine patients with low back pain which has been reported by our group previously [6] are not surprising. It may even be questioned whether the environment of the degenerated IVD in dogs with DLSS is more prone to settling of bacteria originating from low grade urogenital infections or that bacteria indeed play a much more important role as the initiating factor in the process of IVD degeneration in dogs.

Distraction of the IVD space results in widening of the foramina and thereby results in indirect decompression of the exiting L7 spinal nerves, it will limit motion and permit fusion [18]. Moreover, distraction can normalize disc height and pressure [53]. A combination of spinal fixation through PSRF and distraction without concurrent discectomy could potentially show a beneficial effect on stability and IVD physiology in dogs, as is seen in human patients suffering from end stage knee osteoarthritis. After two months of applied distraction of the knee joint, clinical improvement and the formation of cartilage-like tissue in the distracted knee were evident for at least two years [54]. In the current study, PSRF in combination with discectomy and distraction was performed in four dogs. Postoperative radiography showed successful distraction in all four cases. In three dogs the LS joint remained distracted for at least six months postoperatively. The fourth dog became very painful three days after surgery and radiography showed collapse of the L7-S1 IVD space. The dog was treated with pain medication and clinical signs resolved. This case demonstrates that distraction alone with PSRF in dogs with severe DLSS exerts strain on the interface between bone and pedicle screws and this may be solved by the use of an vertebral interbody cage. In dogs with caudal cervical spondylomyelopathy, a combination of vertebral stabilization and intervertebral implants tend to be more effective in gaining bony fusion and can also maintain distraction [39]. In spinal surgery in human patients with low back pain, intervertebral cages are also frequently used (with or without vertebral stabilization) [55–57]. Aggressive abrasion of the end plates together with a spinal cage may also promote spinal fusion. The use of a cage as a stand-alone-device or in combination with PSRF (and the effect on spinal fusion) needs to be investigated in future studies.

## Conclusions

PSRF can be an effective therapy option for dogs with severe DLSS disease with or without radiological evidence of discospondylitis, in which no other treatment is available. PSRF alone does not result in interbody vertebral bone fusion between L7 and S1.

## Methods

### Dogs

Twelve dogs with DLLS treated by PSRF were included in this retrospective study. The medical records of the dogs were systematically reviewed and the signalment, clinical history, findings on clinical examination, force plate data, radiographic, and CT- and/or MR imaging were retrieved. Due to the retrospective nature of the current study, no ethical approval was required. The owners consented to the use and disclosure of patient- and questionnaire data for the current study. Table 1 shows the signalment and clinical history of all the dogs included in this study.

### Clinical examination

All dogs underwent a full clinical examination, consisting of a general physical, orthopedic and neurological examination by a board-certified veterinary surgeon (BPM). Neurological deficits were graded based on the scale used by Griffith (modified by Sharp and Wheeler 2005): grade 0 (normal), grade 1 (spinal pain only), grade 2 (ambulatory paraparesis), grade 3 (non-ambulatory paraparesis), grade 4 (paraparalysis with deep pain perception), and grade 5 (paraparalysis without deep pain perception) (Table 2).

### Diagnostic imaging

Ventrodorsal and lateral radiographic views were obtained with the LS spine in neutral position. CT- and MRI-scans were obtained under general anesthesia and dogs were positioned in sternal recumbency with the pelvic limbs extended caudally. CT-scans were obtained with a third-generation CT-scanner^1^. Contiguous 2-mm-thick slices were acquired. MRI was performed with a 0.2 Tesla open magnet^2^. Contiguous 3-mm-thick sagittal T1- and T2-weighted images and transverse T1- and T2-weighted MR images were obtained.

Pre-operatively, CT-scans and/or MRI scans were performed. The acquired diagnostic images were evaluated by a board-certified radiologist, a board-certified orthopedic surgeon (BPM), and a PhD student/DVM (ART). During surgery, correct position of the screws and the amount of distraction was verified by fluoroscopy. Post-operatively, the position of the pedicle screws, the amount of bony fusion and the development of ASP were recorded by radiography or CT on several occasions. In four dogs manual distraction was applied, and the amount of distraction was calculated by comparing the disc height indices prior to treatment with the PSRF device in place. The disc height index was calculated on the radiographs and midsagittal CT reconstructions by using the method described by Hoogendoorn et al. [58]. Imaging performed during follow up visits is summarized in Table 3.

### Force plate analysis (FPA)

Ground reaction forces (GRFs) were measured using a quartz crystal piezoelectric force plate^3^ together with the Kistler 9865E charge amplifiers. The force plate itself was 60 cm wide and 40 cm long, and was mounted flush with the surface in the center of an 11 m long walkway. The middle 5 m of the runway was bordered by a 50-cm high fence to guide the dogs over the force plate. GRFs were measured by force transducers, which were located in every corner of the plate. The amplifiers were connected to an analog-digital converter, interfaced with a computer that stored the signals. The sampling rate was 100 Hz. The signals corresponded with the GRFs in the mediolateral (Fx), craniocaudal (Fy) and vertical (Fz) direction. The Fz was calibrated with a standard weight before each recording session. Forward velocity of the dog was measured during FPA, using two photoelectric switches spaced 3 m apart and centered on the force plate and computer timing. FPA recordings were automatically started and stopped by these photoelectric switches. All dogs were guided over the force plate on a leash at a walking gait with an average speed of 1.08 m/s (standard deviation 0.08 m/s). Data recorded from measurements in which a thoracic limb and, after a short interval, the ipsilateral pelvic limb contacted the plate were considered valid. A minimum of eight recordings were used for data processing. All forces were normalized for body weight. Ratios between pelvic (P) and thoracic (T) limbs were calculated: P/T Fy-, P/T Fy + and P/T Fz+. +. Obtained results were compared to previous FP results in normal dogs and dogs with low back pain [19].

### Surgical procedure and postoperative care

All dogs were operated by the same ECVS board-certified surgeon (BPM). All dogs underwent a dorsal laminectomy [2] and several additional procedures before PSRF depending on the imaging and surgical findings (Table 2). Discectomy yielded nucleus pulposus (NP) material that was cultured for bacteria in 10/12 dogs. The spinous processes of L7 and S1 and the lamina of L7 were preserved to serve as autologous bone transplant in ten dogs. In one dog (case 4) a cancellous bone transplant was obtained from the iliac crest. The bone chips and cancellous bone were packed into the intervertebral disc space up to 5 mm beneath the floor of the vertebral canal. An autologous fat transplant, harvested from free subcutaneous tissue, was placed ventral to the cauda equina, and a larger piece was deposited dorsally in the laminectomy site with the aim of preventing dural adhesions and new bone formation [2].

In one dog the compression was severely lateralized necessitating a unilateral facetectomy (dog 5), in two other dogs bilateral facetectomy was necessary (dog 8 and 10). In dog 5 the left S1 nerve had an abnormal appearance and was completely resected and sent for histology.

Thereafter, PSRF was performed as described by Smolders et al. [14]. Briefly, the entry points of L7 and S1 were identified and the corridors in the cancellous bone within the pedicle were prepared using a bone awl and probe^4^. Once the ventral cortex was reached, the pedicle probe was removed from the screw corridor. To facilitate screw anchorage in the ventral vertebral cortex, predrilling of the ventral cortex was performed with a K-pin (1.2 mm). Four 25 mm long, 4 mm wide titanium pedicle screws^d^ were inserted into the pedicle and vertebral body. Two 5 cm long, 6 mm wide titanium rods were used to connect the L7 pedicle screw with the ipsilateral S1 pedicle screw. The rod was slightly adjusted with a rod bender to acquire a proper fit on both screw heads. Once a tight fit was obtained, the sleeves and nuts were applied and tightened. Optimal screw anchorage was achieved by involving both the medial and lateral pedicle cortex. “Cortical encroachment” was identified when the pedicle cortex could not be visualized or as “frank penetration” when the screw was outside the pedicular boundaries [59–61]. Screw placement was considered optimal when screws involved the cortical bone and not fully penetrated the ventral vertebral cortex. Intraoperative fluoroscopy was used to verify correct placement of the screws. Four dogs underwent manual distraction as well because of intervertebral foraminal stenosis evident on pre-operative imaging. Manual distraction was applied to the base of the pedicle screws using a Gelpi retractor followed by tightening the screw heads to the rods. The amount of distraction was estimated based on the mobility of the LS segment and did not exceed 5 mm. Postoperative care consisted of leash restraint and exercise restriction for a period of six weeks and after that, the dogs were allowed to gradually return to their normal exercise regime within three months after surgery.

### Follow-up and questionnaires to owners

Follow-up data were collected from the medical records, by using questionnaires [6, 13, 19] to owners, by interviewing the owners and by reexamination of the dogs. Questionnaires for follow-up evaluation (Table 5) were sent to all owners of dogs that had undergone PSRF within the last four years. Two dogs were lost in follow-up due to unrelated mortalities. The questionnaires included questions regarding the history, clinical signs before surgery and the owner’s satisfaction with the outcome at three months and one year after surgery.

**Table 5:** Questionnaire to the owners of dogs before, at three months and more than one year after pedicle screw-rod fixation for degenerative lumbosacral stenosis

Types	Questions
YES or NO questions	Did the symptoms disappear after surgery?
	Did the symptoms recur after surgery (after an initial improvement)?
Open questions	How is your dog after surgery?
	Does your dog refuse certain movements?
	Did your dog receive further treatment after surgery?
Questions with a 10-point scale	Does your dog have pain in the pelvic limbs and shows lameness?
	Does your dog show weakness in the pelvic limbs?
	Does your dog have low back pain?
	Does your dog have difficulty rising up?
	Does your dog have difficulty lying down?
	How would you rate muscle volume in the pelvic limbs of your dog?
	How is your dog holding its tail?
	Is your dog able to wag its tail?
	Does your dog show loss of control of urination and defecation?
	Does your dog show pain when you touch the lower back?

Statistical analysis was performed using software (SPSS 22 for Windows; SPSS Inc., Chicago, IL). Normal distribution of the data was checked by performing the Shapiro Wilks test. The reliability of the responses to the questionnaires was tested by calculation of Cronbach’s α where a value of >0.70 was considered reliable [62]. Comparison of the mean scores of the questionnaires before surgery, at 6 months, and >1 years after surgery was conducted using the Friedman’s test. If there was a significant difference (*P* < 0.05), post hoc tests were performed for each time point. The pre-operative Griffith score for neurological (dys)function was compared to the Griffith score appointed at the last follow up visit using the Wilcoxon signed rank test. Significance was set at *P* < 0.05.

## Abbreviations

ASP: adjacent segment pathology
BMP: bone morphogenetic protein
CT: computed tomography
DLSS: degenerative lumbosacral stenosis
FPA: force plate analysis
IVD: intervertebral disc
LS: lumbosacral
MRI: magnetic resonance imaging
NP: nucleus pulposus
NSAID: non-steroidal anti-inflammatory drug
PSRF: pedicle screw-rod fixation

## Footnotes

1 Tomoscan CX/S, Philips.

2 Magnetom Open Viva, Siemens.

3 Kistler type 9261, Kistler Instrumente.

4 USS Small Stature, DePuy Synthes.
